# A Hub and Spoke Learning Program in Bariatric Surgery in a Small Region of Italy

**DOI:** 10.3389/fsurg.2022.855527

**Published:** 2022-03-24

**Authors:** Antonio Buondonno, Pasquale Avella, Micaela Cappuccio, Andrea Scacchi, Roberto Vaschetti, Giancarlo Di Marzo, Pietro Maida, Claudio Luciani, Bruno Amato, Maria Chiara Brunese, Daniela Esposito, Lucio Selvaggi, Germano Guerra, Aldo Rocca

**Affiliations:** ^1^General Surgery Unit, A. Cardarelli Hospital, Campobasso, Italy; ^2^Department of Medicine and Health Science, University of Molise, Campobasso, Italy; ^3^General Surgery Unit, Ospedale del Mare, Centro Sanitario Locale Napoli 1 Centro, Naples, Italy; ^4^Department of Clinical Medicine and Surgery, University of Naples Federico II, Naples, Italy; ^5^Department of Internal Medicine and Clinical Nutrition, Institute of Medicine, Sahlgrenska Academy, University of Gothenburg and Department of Endocrinology, Sahlgrenska University Hospital, Gothenburg, Sweden; ^6^Department of Advanced Medical and Surgical Sciences, Università degli Studi della Campania “Luigi Vanvitelli”, Naples, Italy

**Keywords:** bariatric surgery, Hub and Spoke, sleeve gastrectomy, BioEnterics Intragastric Balloon, metabolic surgery, obesity

## Abstract

**Background:**

Metabolic and bariatric surgery (BS) are considered life-changing and life-saving treatments for obese patients. The Italian Society of Obesity Surgery (SICOB) requires at least 25 operations per year to achieve the standard of care in the field. Despite the increasing need to treat obese patients, some small southern regions of Italy, such as Molise, do not have enough experience in bariatric procedures to be allowed to perform them. Therefore, our aim was to run a Hub and Spoke Program with a referral center in BS to treat obese patients and provide a proper learning curve in BS in Molise.

**Methods:**

In 2020, the “A. Cardarelli Hospital” in Campobasso, Molise, started a formal “Learning Model of Hub and Spoke Collaboration” with the Hub center “Ospedale Del Mare”, Naples. A multidisciplinary approach was achieved. Patients were supervised and operated under the supervision and tutoring of the referral center. We retrospectively reviewed our prospectively collected database from February 2020 to August 2021 in order to analyze the safety and effectiveness of our learning program.

**Results:**

In total, 13 (3 men and 10 women) patients underwent BS with the mean age of 47.08 years and a presurgery BMI of 41.79. Seven (53.84%) patients were the American Society of Anesthesiologist (ASA) II, and 6 (46.16%) patients were ASA III. Twelve (92.31%) procedures were laparoscopic sleeve gastrectomies, 1 (7.69%) patient underwent endoscopic BioEnterics Intragastric Balloon (BIB) placement. One (8.33%) sleeve gastrectomy was associated to gastric band removal. Mean surgical time was 110.14 ± 23.54 min. The mean length of stay was 4.07 ± 2.40 days. No Clavien-Dindo ≥ III and mortality were reported. The follow-up program showed a mean decrease of 11.82 in terms of body mass index (BMI) value. The last 5 procedures were performed by the whole equips from “A. Cardarelli” under external tutoring without any impact on complication rate.

**Conclusion:**

The setup of a proper Hub and Spoke Program may allow to perform BS to provide the standard of care. This approach may reduce health costs and related patient migration.

## Introduction

The WHO has estimated that 1.9 billion adults worldwide are overweight and 650 million are obese (1). In Italy, as reported by Global Obesity Observatory, the overall percentage of patients with body mass index (BMI) > 30 kg/m^2^ is across 12 and 10% for men and women over 18 years old, respectively (2). Despite it being a high percentage, it is lower than the mean value of European states (2). In particular, in Molise, a small region of Italy, the overall percentage of obesity is over 14% in both genders: one of the highest national values (3). In literature, it is clearly described a link among obesity and hormonal, endothelial and inflammatory level alterations (4, 5), and pieces of evidence regarding the association between the increased BMI and carcinogenesis (6–8).

The metabolic and bariatric surgery (BS) showed to be the most successful treatment for weight loss and to reduce the patients' comorbidities due to obesity (9, 10).

Bariatric surgery and many other elective surgical services had to deal with the widespread postponements in many parts of the world during the Coronavirus Disease-2019 (COVID-19) pandemic (11, 12). Nevertheless, the surgical treatment of obesity cannot be defined as “*elective”* (13), because nowadays it is considered a life-changing intervention and a life-saving surgery, improving health, quality of life, and long-term survival (11).

Therefore, “A. Cardarelli Hospital” in Campobasso (Molise) started a “Teaching/Learning Model of Hub and Spoke Collaboration” among some referral centers for bariatric, colorectal, and liver surgery (14), in order to reduce patient migration offering the best standard of care to people for all the surgical specialties. The learning programs allow to guarantee effective treatment and safety procedures in patients with morbid obesity also during the critical pandemic period as reported in minimally invasive approaches performed in complex surgery (15–18).

Hub and Spoke Programs have already demonstrated a great impact on regional health programs avoiding health migration, reducing costs, and decreasing the waiting times for surgery (15, 19, 20).

Our study aimed to evaluate the safety and effectiveness of the Hub and Spoke Bariatric Learning Program in a small Italian region analyzing all the peri-, intra-, and postoperative outcomes and the BMI reduction, Total Weight Loss (%TWL), and Excess Weight Loss (%EWL) after 30 and 90 days from surgical procedures, in order to reduce the health system costs and patients migration.

## Materials and Methods

### Hub and Spoke Program

Due to the limited number of inhabitants, Molise does not offer a formal plan specialized in the treatment of obese patients. Consequently, the General Surgery Unit of “A. Cardarelli Hospital”, in Campobasso, Italy, started a partnership with the BS unit of “Ospedale del Mare”, Naples, Italy, directed by Prof. Pietro Maida.

Following the BS guidelines, provided by the Italian Society of Obesity Surgery (SICOB) (21), a multidisciplinary team (MDT) has been setup. Bariatric surgeons, dieticians, nutritionists, psychologists, and anesthetists collaborate and discuss all the cases.

All patients were operated under the supervision and tutoring of the referral center surgeon. The surgeons involved in the Hub and Spoke Learning Program moved from Molise to Naples one time per month during the learning period to be properly trained before surgery.

### Methods

We retrospectively reviewed our prospectively collected database from February 2020 to August 2021 according to STrengthening the Reporting of OBservational studies in Epidemiology (22). The elective BS was interdicted in the months between March and June 2020 and from November 2020 to May 2021 due to the COVID19 pandemic period in order to reduce in-hospital viral transmission and related postoperative pulmonary complications. The goal was to preserve the hospital workers and to better care for patients affected by severe acute respiratory syndrome coronavirus-2 (SARS-CoV-2) infection, and to have more beds for patients.

We included all obese patients (BMI > 30 kg/m^2^) (23) who underwent BS at “A. Cardarelli Hospital” in that period. No exclusion criteria were chosen. Under the supervision of MDT, all patients underwent a 3-week very low-carbohydrate ketogenic diet program before surgery (10). Before the admission in the surgery unit, all patients performed a molecular rhino-pharyngeal swab to verify the negativity to SARS-CoV-2 infection. According to SICOB guidelines, all patients, before surgery, performed dietary and psychological evaluation, routine blood samples, chest-XR and ECG, and esophagogastroduodenoscopy (EGDS). All patients carried out an oral glucose tolerance test (OGTT) and glycosylate hemoglobin test (HbA1c).

The serum levels of triglycerides, low-density lipoprotein (LDL) cholesterol, high-density lipoprotein (HDL) cholesterol, and total cholesterol were measured on a preoperative day, subsequently at 90 days after surgery.

In selected cases, spirometry, echocardiogram, and peri-operative Continuous Positive Airway Pressure (CPAP) were performed. The intraoperative risk was evaluated with the American Society of Anesthesiologist (ASA) score (24).

An Enhanced Recovery After Surgery (ERAS) program was used to achieve a rapid recovery of patients' conditions (25–27).

Postoperative complications were assessed according to the Clavien-Dindo classification (28). Follow-up was planned at 30 and at 90 days after surgery.

Due to the COVID-19 pandemic, telemedicine has been used in some cases to perform a follow-up and prescribing therapies by means of communication technologies (29).

All individuals included in this study signed informed consent for the scientific anonymous use of clinical data. The study was conducted according to the guidelines of the Declaration of Helsinki and approved by the Institutional Review Board of the University of Molise (protocol number 10/21, approved date: May 12, 2021).

### Technical Notes

#### Sleeve Gastrectomy

Antibiotic prophylaxis was performed 30 min before intervention [ceftriaxone 2 g intravenous (i.v.)]. No patients presented allergy to the prophylactic regimen. Due to the intimate correlation between obesity and thrombotic risk, all patients were subjected to antithrombotic therapy (30, 31). All operations were performed through a minimally invasive approach under general anesthesia. A nasal-gastric tube was placed after anesthesia, and it was removed on postoperative day 3. A urinary catheter was placed according to the expected procedure length.

The tutor, and operating surgeon, stood to the patient's right, the assistant on the left side. The abdomen was insufflated to 12 mmHg to achieve pneumoperitoneum, and 5 ports were located.

We used the reverse Trendelenburg position to facilitate the fall of the transverse colon and small intestine toward the pelvis. We did a complete mobilization of the greater curvature of the stomach proximally to His' angle. After identification of pylorus, the first operating surgeon identified the site of transection 5–6 cm proximal to the pylorus. We conducted the dissection along the greater curvature at the stomach mid-body. A linear stapler was used to complete the dissection, after the introduction of a blunt-tipped bougie dilator (32). To avoid technical drawbacks, methylene blue dye was used to perform a leak test during surgery. The peritoneal drainage tube was inserted, and it was removed when the peritoneal drainage volume was <20 ml/die.

During the procedure, we preserved the splenic vessels and avoided an extreme splenic traction. Postoperative Nausea and Vomiting (PONV) and prophylaxis were performed using double metoclopramide (10 mg/2 ml) injections (33). A liquid diet was ongoing on postoperative day 3. After 2 weeks, patients were encouraged to eat a semi-solid diet and were progressively advanced with a normal diet over the following 2–4 weeks.

In the absence of clinical signs of the leak, stenosis, and other complications, we scheduled discharge.

#### BioEnterics Balloon Placement

After patients' sedation with midazolam or propofol, we performed an upper gastrointestinal endoscopy in order to exclude eventual pathologies. The BioEnterics Balloon (BIB) insertion and the postoperative treatment were performed according to other experiences available in the literature (34). A liquid diet was ongoing on postoperative day 3, solid on postoperative day 10.

Follow-up was scheduled 1 week after BIB positioning and every 3 weeks for a 6-month period. At the end of 6 months, after sedation, we removed the BIB through a single-channel endoscope and dedicated device.

### Statistical Analysis

All quantitative data are reported as mean ± SD.

The difference between preoperative BMI, 30 days and 3-month BMI from surgery was analyzed to evaluate the success of the surgery. Weight loss was also calculated as %TWL during follow-up. The %TWL value was estimated through the formula: [(initial weight – current weight)/(initial weight)] × 100.

The excess weight loss (%EWL) was estimated using the formula: (weight loss/baseline excess weight) × 100, the weight loss is the preoperative weight – initial weight loss. The baseline excess weight is represented by the initial weight – ideal weight (X), and where X = 25 × m^2^. An ideal BMI (25 kg/m^2^) was used to calculate the X.

A two-tailed *p* < 0.05 was established as statistically significant. IBM Statistical Package for the Social Sciences (IBM SPSS®) was used to analyze data.

## Results

A total of 13 (3 men and 10 women) patients who were included in our study underwent BS between February 2020 and August 2021.

The mean age was 47.08 years ± 7.54 with a mean BMI, before surgery, of 41.79 ± 6.02.

Regarding ASA score 7 (53.84%), patients were ASA II and 6 (46.16%) patients were ASA III.

The most frequent comorbidities were hypertension (84.81%), gastritis (84.61%), and diabetes mellitus (30.76%). No patient was found positive at SARS-CoV-2 molecular swab. Baseline characteristics of patients are depicted in Table 1.

**Table 1:** Baseline characteristics of patients.

**Variables**	**N. (%) and/or Mean ±SD**
**Age** (**years**)	47.08 ± 7.54
**Gender**	
Male	3 (23.08)
Female	10 (76.92)
**ASA**	
II	7 (53.84)
III	6 (46.16)
**Comorbidities**	
Hypertension	11 (84.61)
Gastritis	11 (84.61)
Diabetes Mellitus	4 (30.76)
Hypothyroidism	3 (23.08)
Esophagitis	2 (15.38)
**SARS-CoV-2 swab positivity**	0 (0)

*SD, standard deviation*.

All procedures were performed laparoscopically. Twelve (92.31%) procedures were sleeve gastrectomies (LSG) and 1 (7.69%) patient underwent endoscopic BIB placement. One (8.33%) LSG was associated to gastric band removal.

All surgical operations were performed under general anesthesia, except for the placement of BIB.

Mean surgical time was 110.14 ± 23.54 min. No hiatal hernia was found or repaired. No Intensive Care Unit (ICU) admission was reported.

Clavien-Dindo I-II complications were observed in 4 out of 13 patients: we reported 3 (23.07%) cases of PONV and 1 (7.69%) patient, after LSG, required Total Parental Nutrition (TPN) during hospitalization.

The mean length of stay was 4.07 ± 2.40 days. No mortality was reported.

Surgical characteristics and postoperative course are shown in Tables 2, 3.

**Table 2:** Intraoperative and postoperative course.

**Variables**	**N. (%) and/or Mean ±SD**
**Intraoperative course**	
**Surgical approach**	
VLS	12 (92.31)
EGDS	1 (7.69)
**Type of surgery**	
LSG	12 (92.31)
BIB	1 (7.69)
**Associated procedures to LSG**	
Gastric band removal	1 (8.33)
**Mean operative time (minutes)**	110.14 ± 23.54
**Postoperative course**	
**Clavien-Dindo classification**	
I	3 (23.07)
II	1 (7.69)
≥III	0 (0)
**TPN**	1 (7.69)
**PONV**	3 (23.07)
**Length of stay (days)**	4.07 ± 2.40

*SD, Standard Deviation; VLS, Video-laparoscopy; EGDS, esophagogastroduodenoscopy; LSG, Laparoscopic Sleeve Gastrectomy; BIB, BioEnterics Intragastric Balloon; TPN, Total Parenteral Nutrition; PONV, Postoperative Nausea and Vomiting*.

**Table 3:** Outcomes after 90 days from BS.

	**Pre-operative measurement,** **Mean ±SD**	**90-day post-operative measurement,** **Mean ±SD**	***P*-value**
N. of patients	13	13	
Weight (kg)	112.22 ± 22.48	80.47 ± 16.91	**0.0004**
Height (cm)	164.00 ± 9.07		
BMI (kg/m^2^)	41.79 ± 6.02	29.97 ± 4.44	**<0.0001**
**Serum lipid** **profile**			
Total cholesterol (mmol/L)	194.22 ± 21.34	173.82 ± 18.57	**0.0157**
LDL (mmol/L)	130.00 ± 21.12	118.30 ± 16.32	0.1271
HDL (mmol/L)	39.57 ± 6.54	41.56 ± 5.49	0.4090
Triglyceride (mmol/L)	120.44 ± 26.00	92.73 ± 29.12	**0.0172**
**Other** **parameters**			
Glycemia (mg/dL)	107.36 ± 42.89	102.42 ± 31.75	0.7414
Hb (g/dL)	13.15 ± 1.21	12.81 ± 1.67	0.5578
eGFR^*^ (ml/min/1.73 m^2^)	103.05 ± 12.65	102.71 ±10.09	0.9402

*SD, Standard Deviation; BMI, Body Mass Index; LDL, low-density lipoprotein; HDL, high-density lipoprotein; Hb, hemoglobin; eGFR, estimated Glomerular Filtration Rate*.

* *eGFR was calculated according to CKD-EPI formula. Bold values are statistically significant findings*.

The follow-up rates were 100% at 30 and 90 days.

**Figure 1:**
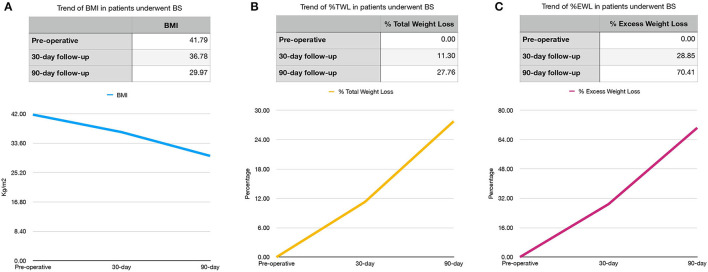
Preoperative, 30- and 90-day BMI **(A)** and %TWL **(B)** and %EWL **(C)** trends in bariatric surgery (BS) patients. BMI, body mass index (kg/m^2^); %TWL, Total Weight Less; %EWL, Excess Weight Loss.

BMI, %TWL, and %EWL trends are shown in Figure 1.

## Discussion

Our study demonstrates the safety and the efficacy of the bariatric procedures, mainly LSG, performed in a peripheric center when involved in a Hub and Spoke Program.

Analyzing our results concerning preoperative patient characteristics, they are superimposable to other relevant casistics from referral centers for BS (35). All the patients have at least one comorbidity in addition to obesity. The most common comorbidities were hypertension and diabetes as reported in the literature (36–39).

Patients who underwent BS in our center were all ASA II and III. It is clear that high-volume centers can report more variability patients' ASA scores due to the greater number of cases (40, 41). The absence of ASA I patients might influence the postoperative data analysis.

Concerning intra-operative courses, all the procedures were performed through a laparoscopic approach following the standard of care for referral bariatric centers in the USA (42). No conversion or re-intervention is reported.

It can also be speculated that the number of the complications reported is higher than reported by a referral center (30 vs. 5–15%, respectively) (43, 44), but we shall underline that we reported only Clavien-Dindo I or II complications treated in conservative approach.

Furthermore, the small sample size influenced the complication rate. No patient presented the complications reported as serious as an anastomotic leak, cardiac, genitourinary, hemorrhagic, neurologic injuries, obstruction, postoperative shock, pulmonary, splenic injury, thromboembolic event, wound infection, and reoperation (42, 45–48). No patients needed ICU stay.

Moreover, this finding may benefit from the small sample size, but it is also due to a careful selection of cases, which were always discussed with the Hub MDT.

Moreover, a proper step by step learning curve of the whole team was established to achieve the best results as described in Vitiello et al.'s experience (35).

The mean hospital stay is higher than the length of stay (LOS) reported in the literature for a high-volume center for LSG (35, 49–51). As known, LOS may be influenced by modifiable and non-modifiable factors (52).

In our case, most of the factors that affect LOS cannot be modified, such as age, BMI, ASA, and creatinine.

As reported by Tholey et al. (53), the ASA score > 2 was a significant predictor of an LOS longer than 48 h, probably due to the greater risk of even mild complications.

Among the non-modifiable factors, there are also the socio-economic conditions and the geographical distance between little towns and the Cardarelli Hospital.

In Molise, it might be difficult for many patients to undergo 1-day hospital service before and after surgery, forcing them to hospitalize these patients and frail and lonely patients (54).

We are aware that center volume correlates to results (55–58), but in a moment in which health migration constitutes a risk for the population due to the COVID19 pandemic (59, 60), a Hub and Spoke Program for elective BS may offer patients the chance to be treated in the safest and most effective way without the costs and risks of health migration (20).

The chance to be treated in their own region is also important for the families of the patients because in the COVID19 pandemic period, it was even more difficult to assist patients far from home (61).

Therefore, we can affirm that our approach has led us to guarantee effective treatment and safety procedures also during the critical pandemic period, as reported by Bonalumi et al. in cardiac and vascular surgery during the COVID19 pandemic (62). Moreover, Ceccarelli et al. have been experienced the safety of this program in liver surgery and concluded that it may allow patients to undergo a suitable standard of care for complex surgery (20).

Furthermore, our Hub and Spoke Learning Program aims precisely to improve the capacity and experience of the surgical team in order to best manage all the modifiable factors reported by Meneveau et al. with a consequent reduction of complications and hospital stay (52).

During the follow-up, our patients had a consistent reduction in BMI and our findings were in line with results from referral centers for BS (35, 51, 63).

Our aim is to share the first report of a successful Hub and Spoke Program, which allowed to best manage patients from a small region of Italy, where it is very difficult to reach all the standards of care for the most frequent surgical pathologies, but where health migration should be reduced respecting the rules of the best surgical practice.

### Limitations

The major drawback of our study is the small number of patients enrolled in the study. However, we would like to share our successful experience to encourage the application of Hub and Spoke Programs, which are up to now the best way to reduce health mobility and consequent health costs for patients coming from small regions achieving the best standard of care.

## Conclusion

Our pilot study has the aim to demonstrate the effectiveness of Hub and Spoke Learning Program to reduce migration and costs ensuring the standard of care in BS, especially in laparoscopic sleeve gastrectomy. Our program is still being continued and we are enrolling even more patients which can undergo BS for the first time in their region. Our further goals are represented by the improvement of outcomes, even more autonomous patient management and the possibility to propose all types of interventions in our hospital.

## Data Availability Statement

The raw data supporting the conclusions of this article will be made available by the authors, with undue reservation.

## Ethics Statement

The studies involving human participants were reviewed and approved by University of Molise. The patients/participants provided their written informed consent to participate in this study.

## Author Contributions

AB, PA, and AR: conceptualization. PA and AR: methodology and writing—review and editing. PA, MC, and RV: software. PA, MC, AS, RV, MB, and AR: validation, writing—original draft preparation, and visualization. MC, AS, RV, GD, GG, CL, MB, DE, and LS: formal analysis and investigation, resources, and data curation. PM, BA, GG, and AR: supervision. All authors have read and agreed to the published version of the manuscript.

## Conflict of Interest

The authors declare that the research was conducted in the absence of any commercial or financial relationships that could be construed as a potential conflict of interest.

## Publisher's Note

All claims expressed in this article are solely those of the authors and do not necessarily represent those of their affiliated organizations, or those of the publisher, the editors and the reviewers. Any product that may be evaluated in this article, or claim that may be made by its manufacturer, is not guaranteed or endorsed by the publisher.
